# Optimization of CRISPR/Cas System for Improving Genome Editing Efficiency in *Plasmodium falciparum*

**DOI:** 10.3389/fmicb.2020.625862

**Published:** 2021-01-08

**Authors:** Yuemeng Zhao, Fei Wang, Changhong Wang, Xiaobai Zhang, Cizhong Jiang, Feng Ding, Li Shen, Qingfeng Zhang

**Affiliations:** ^1^Research Center for Translational Medicine, Key Laboratory of Arrhythmias of the Ministry of Education of China, East Hospital, Tongji University School of Medicine, Shanghai, China; ^2^Key Laboratory of Spine and Spinal Cord Injury Repair and Regeneration of Ministry of Education, Orthopaedic Department of Tongji Hospital, Tongji University, Shanghai, China; ^3^Shanghai Key Laboratory of Signaling and Disease Research, The School of Life Sciences and Technology, Tongji University, Shanghai, China; ^4^Department of Microbiology and Immunology, School of Basic Medical Sciences, Wenzhou Medical University, Wenzhou, China

**Keywords:** Malaria, *Plasmodium falciparum*, CRISPR/Cas9, gene editing, Cpf1

## Abstract

Studies of molecular mechanisms and related gene functions have long been restricted by limited genome editing technologies in malaria parasites. Recently, a simple and effective genome editing technology, the CRISPR/Cas (clustered regularly interspaced short palindromic repeats/CRISPR-associated) system, has greatly facilitated these studies in many organisms, including malaria parasites. However, due to the special genome feature of malaria parasites, the manipulation and gene editing efficacy of the CRISPR/Cas system in this pathogen need to be improved, particularly in the human malaria parasite, *Plasmodium falciparum*. Herein, based on the CRISPR/Cas9 system, we developed an integrating strategy to generate a Cas9i system, which significantly shortened the time for generation of transgenic strains in *P. falciparum*. Moreover, with this Cas9i system, we have successfully achieved multiplexed genome editing (mutating or tagging) by a single-round transfection in *P. falciparum*. In addition, we for the first time adapted AsCpf1 (*Acidaminococcus* sp. Cpf1), an alternative to Cas9, into *P. falciparum* parasites and examined it for gene editing. These optimizations of the CRISPR/Cas system will further facilitate the mechanistic research of malaria parasites and contribute to eliminating malaria in the future.

## Introduction

Malaria remains a major public health threat around the world. In 2018, it was estimated that approximately 228 million cases of malaria occurred worldwide (WHO, 2019). Among the five malarial species infecting humankind, *Plasmodium falciparum* accounts for the most severe malaria cases in African countries. *Plasmodium* parasites harbor a complex life cycle, which is subject to precisely spatiotemporal gene regulation (Philip and Waters, 2015). Since the genome sequencing of *P. falciparum*, the study of gene functions became a hot topic, with focus turned toward the underlying molecular mechanisms of immune evasion, pathogenesis, transmission, and drug resistance. To eliminate the global risk of malaria, basic research of the gene function and molecular mechanism of *Plasmodium* must be expanded.

Many kinds of genetic manipulation tools have been developed for basic research, from conventional homogenous crossover to ZFN (zinc finger nuclease), TALEN (transcription activator-like effector nuclease), and CRISPR/Cas9, making genome editing more convenient and efficient. CRISPR/Cas system is a kind of microbial adaptive immune system that protects the microbe against foreign genetic elements (Jinek et al., 2012). Based on the configuration of effector modules, CRISPR/Cas systems are classified into two classes and six types: Class 1 (Types I, III, and IV) system utilizes multi-protein complexes, whereas class 2 (Types II, V, and VI) system employs a single-component effector protein such as Type II-A Cas9 or Type V-A Cpf1 (van der Oost et al., 2014; Wright et al., 2016).

Recently, the CRISPR/Cas9 system, commonly consisting of a *Streptococcus pyogenes* Cas9 ortholog (SpCas9) and a targeting single guide RNA (sgRNA) (Cong et al., 2013; Mali et al., 2013a), has been used for genome editing in various organisms (Mali et al., 2013b; Zhang et al., 2014; Barrangou and Doudna, 2016), including the malaria parasite *P. falciparum* (Wagner et al., 2014; Kuang et al., 2017). In *P. falciparum*, genome editing with the CRISPR/Cas9 system usually requires co-transfection of two episomal plasmids carrying the Cas9 endonuclease and the sgRNA, along with donor DNA (Ghorbal et al., 2014). Due to the extremely low efficiency of transfection, the co-transfection of two constructs largely limits the efficiency of genome editing in *P. falciparum*. Apart from the widely used Cas9, some Cpf1 orthologs, such as AsCpf1 (*Acidaminococcus* sp. Cpf1), are also demonstrated to mediate efficient genome editing in mammalian cells (Zetsche et al., 2015a, 2017; Gao et al., 2017), but they have not been adapted into malaria parasites so far. Cpf1 has several advantages for genome editing in malaria parasites compared to Cas9. For example, Cpf1 only needs one single CRISPR RNA (crRNA), while Cas9 needs both crRNA and trans-activating crRNA (tracrRNA). Cpf1 can also process its own crRNA array into mature crRNAs, which offers great convenience for multiplexed genome editing. Additionally, the target DNA of Cpf1 is cut into staggered fragments distal to a 5′ T-rich protospacer-adjacent motif (PAM), while the target DNA of Cas9 is cut into blunt ends adjacent to a NGG PAM. The sticky ends and T-rich PAM of Cpf1 will help tremendously in editing the AT-rich genome in malaria parasites.

To optimize the traditional episomal CRISPR/Cas9 system (Cas9e for short), in this study, an integrating version was generated to express the Cas9 nuclease stably in *P. falciparum*. This integrative CRISPR/Cas9 system, named Cas9i, has significantly increased the efficiency of genome editing in *P. falciparum.* With this updated system, not only has the generation of transgenic lines been sped up, but multiplexed genome editing, especially multiplexed tagging, has become feasible in *P. falciparum.* Furthermore, Cpf1 is a very efficient alternative to Cas9 in malaria parasites due to its unique biochemical characteristics. Thus, the utilization of this integrating strategy and the CRISPR/Cpf1 system will provide more alternative tools for genome editing in malaria research.

## Materials and Methods

### Plasmids Construction

**FIGURE 1:**
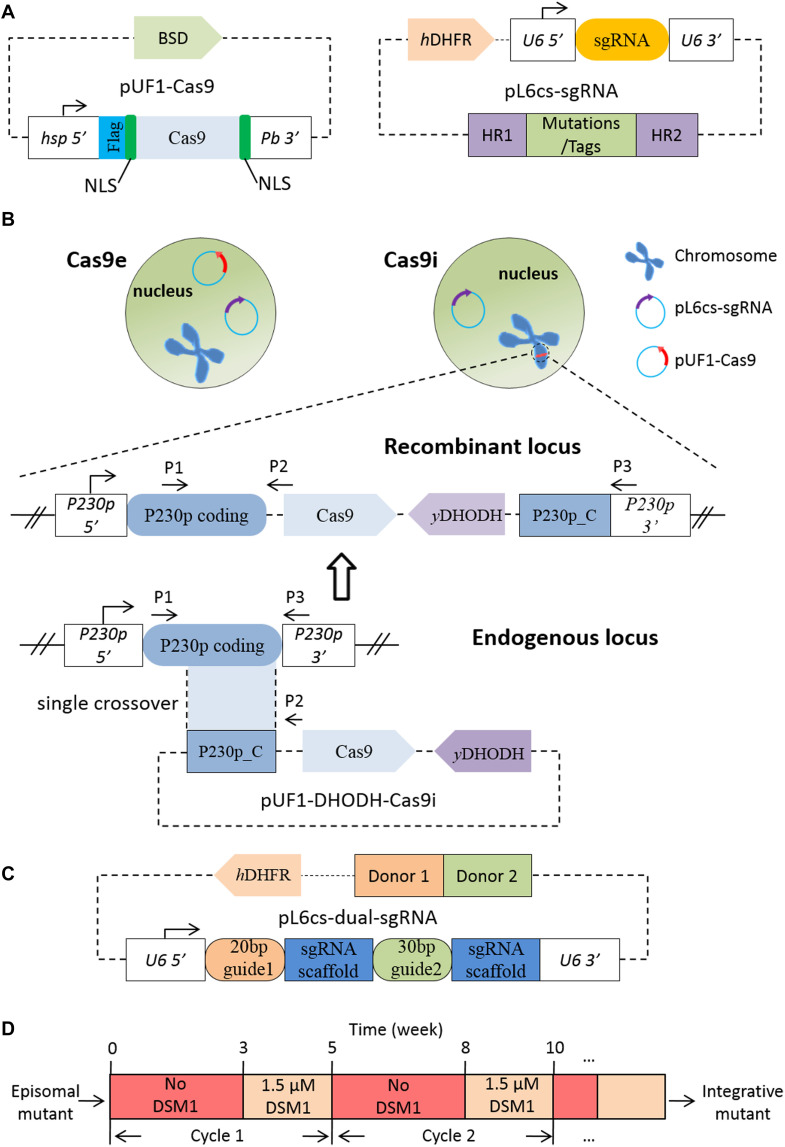
Construction of the Cas9i system and sgRNA array. **(A)** Sketch of *pUF1-Cas9* and *pL6cs-sgRNA*. *pUF1-Cas9* expresses the Cas9 protein with the BSD drug-selectable marker. A flag tag and two NLSs are fused to Cas9 protein. *pL6cs-sgRNA* carries the sgRNA cassette, the donor DNA including the homologous regions (HR1 and HR2) and modifications, and the hDHFR marker. **(B)** Diagram illustrating the Cas9e and Cas9i system. The Cas9e system employs two episomal plasmids in the nucleus while the Cas9i system employs an episomal pL6cs plasmid and an integrated *pUF1-DHODH-Cas9i* plasmid which was integrated into the endogenous *P230p* locus through a single crossover in advance. *pUF1-DHODH-Cas9i* carries the Cas9 expression cassette (the same as *pUF1-Cas9*), yDHODH selection marker, and the C-terminal of *P230p* coding sequence. Primers indicated as P1–P3 were used for verification of integration. **(C)** Sketch of pL6cs bearing the dual-sgRNA array. Multiple sgRNAs are constructed in a single array, consisting of the 20- or 30-bp target sequences following by the 78-bp sgRNA scaffold. Donor DNAs were constructed adjacent to each other. **(D)** Diagram illustrating drug off/on selection cycle. Episomal mutant is cultured for 3 weeks without selection drug (DSM1 in this case), and for another 2 weeks with the drug. This process can be repeated for several cycles.

The plasmids used in our study were constructed based on the *pL6cs-sgRNA* and *pUF1-Cas9* plasmids as described previously (Ghorbal et al., 2014; Figure 1A). *pL6cs-sgRNA* is the vector of sgRNA expression cassette and donor DNA, and expresses *hDHFR* marker for positive selection with WR99210 (Fidock and Wellems, 1997). *pUF1-Cas9* expresses SpCas9 endonuclease, fused with a 3xFLAG tag, two nuclear location sequences (NLS), and the resistance gene of blasticidin S deaminase (BSD). The plasmid *pY010* (*pcDNA3.1-hAsCpf1*) which contains the humanized AsCpf1 coding sequence was ordered from addgene through Beijing Zhongyuan Ltd.

Plasmid *pUF1-DHODH-Cas9i* was constructed to integrate the Cas9 endonuclease into the *P. falciparum* genome, with the selection marker replaced by yDHODH, which is resistant to the selection drug DSM1 (Figure 1B). BSD selection marker was replaced because of the emergence of endogenous resistance to BSD during long-term cultivation. Firstly, we amplified the C-terminal 1219-bp fragment of *PfP230p* gene with primers P20/P21 and cloned it into *pUF1-Cas9* using the restriction sites *Eco*RI and *Avr*II. Next, we replaced the BSD resistance gene cassette with yDHODH gene using the primers P22/P23 at the restriction sites *Nco*I and *Xba*I to generate the final plasmid *pUF1-DHODH-Cas9i*.

To edit the target gene individually, *pL6cs-Sir2B* (transcriptional regulatory protein Sir2B, PF3D7_1451400) and *pL6cs-ARP6* (actin-related protein 6, PF3D7_0719300) were constructed to carry the corresponding sgRNAs and donor DNAs, respectively. The 20-bp guide sequences were designed as two complementary oligonucleotides and cloned into *pL6cs-sgRNA* after annealing using the restriction sites *Avr*II and *Xho*I. The oligonucleotides for Sir2B and ARP6 were P24/P25 and P26/P27, respectively. Then, the donor DNAs were amplified and cloned to the constructs above using the restriction sites *Asc*I and *Afl*II. The primer sets for donor DNAs (homology arms and tags) were P28/P29 and P30/P31 for Sir2B and P32/P33, P34/P35, and P36/P37 for Arp6, respectively. These PCR products were spliced by overlap extension PCR.

To generate the dual-sgRNA arrays for the dual pL6cs constructs, we amplified the fragments bearing the 20-bp first guide sequence, the 78-bp scaffold, and the 30-bp second guide sequence, and cloned them to *pL6cs-sgRNA* to create the dual-sgRNA arrays consisting of two sgRNAs (Figure 1C). The primers for *pL6cs-K13-Sir2B* and *pL6cs-Arp6-ARP4* were P38/P39 and P40/P41, respectively. For the donor DNAs, we amplified four fragments using the primer sets P42/P43, P44/P45, P46/P29, and P30/P31 to generate *pL6cs-K13-Sir2B*; six fragments using the primer sets P32/P33, P34/P35, P36/P47, P48/P49, P50/P51, and P52/P53 were amplified to generate *pL6cs-ARP6-ARP4*. These fragments were spliced respectively by overlap extension PCR and subsequently cloned to the corresponding plasmids side by side at the restriction sites *Asc*I and *Afl*II.

The plasmid *pUF1-Cpf1* was constructed in two steps. In the first step, we generated a middle vector by two sequential PCR amplifications: the first PCR amplified a fragment from *pUF1-Cas9* with primers P54/P55 and the second amplification was conducted using primers P54/P56, with the first PCR product as the template, generating the final Flag-NLS-*Asc*I-*Bam*HI-NLS fragment. This fragment was cloned to *pUF1-Cas9* with restriction sites *Xho*I and *Kpn*I, generating the middle vector. Then in the second step, the fragment of AsCpf1 coding sequence amplified from pY010 using primers P57/P58 was cloned to the middle vector with the restriction sites *Asc*I and *Bam*HI to generate *pUF1-Cpf1*.

To generate *pL6cs-Cpf1-crRNA* that contains direct repeat (DR) of AsCpf1 crRNA, we amplified the U6_5′-DR-*Pst*I-*Xho*I fragment using primers P59/P60 and cloned it to pL6cs-sgRNA with the restriction sites *Nco*I and *Avr*II. The 23-bp guide sequences for AsCpf1 were also designed as two complementary oligonucleotides and cloned to *pL6cs-Cpf1-crRNA* after annealing using the restriction sites *Pst*I and *Xho*I. The oligonucleotides were P61/P62 and P63/P64 for Sir2B and ARP6, respectively. The donor DNAs were cloned to *pL6cs-Cpf1-crRNA* using restriction sites *Afl*II and *Asc*I after the construction of crRNAs. For *pL6cs-Cpf1-Sir2B*, two fragments were amplified with primers P65/P66 and P67/P68 bearing the desired mutations. For *pL6cs-Cpf1-Arp6*, three fragments were amplified with primers P69/P70, P34/P35, and P71/P72 bearing the desired mutations and HA tag. The fragments were spliced respectively by overlap extension PCR.

All PCR reactions were performed using PrimeSTAR^®^ HS DNA Polymerase (R010A, TaKaRa, Japan). All clone reactions were conducted with the ClonExpressII One Step Cloning Kit (C112, Vazyme, China) following the protocols, requiring a 15-bp homology. The constructs were transformed into XL10 competent cells (C503-02, Vazyme, China) and extracted with TIANprep Rapid Mini Plasmid Kit (DP105-03, Tiangen, China). The plasmids were confirmed by restriction enzyme digestion and sequencing. The confirmed constructs were finally isolated with NucleoBond^®^ Xtra Midi Plus (740412.50, MACHEREY-NAGEL, Germany) for transfection.

### Parasite Culture and Transfection

All *P. falciparum* parasites were cultured as previously described (Ghorbal et al., 2014) with 5% O^2^ and 5% CO^2^ at 37°C. Parasites were synchronized with 5% sorbitol solution for ring stage or purified on a 40/70% percoll discontinuous gradient for schizont stage. For transfection, fresh human type O erythrocytes were electroporated in cytomix (120 mM KCl, 10 mM KH_2_PO_4_, 25 mM HEPES, 2 mM EGTA, 0.15 mM CaCl_2_, 5 mM MgCl_2_, pH 7.6) with 100 μg of each plasmid under standard electroporation parameters (Fidock and Wellems, 1997; Fidock et al., 1998) and enriched late schizonts were mixed with the electroporated erythrocytes immediately. For the episomal CRISPR system (Cas9e or Cpf1), the pL6cs constructs were co-transfected with *pUF1-Cas9* or *pUF1-Cpf1* plasmid. For the Cas9i system, firstly *pUF1-DHODH-Cas9i* plasmid was transfected to the *P. falciparum* 3D7 strain as episomes. As soon as the episomal mutant was obtained, it was screened by drug off/on cycles (Figure 1D) to attain the integrative parental mutant generated by a single-crossover recombination event. Then the corresponding pL6cs constructs were transfected into the integrative parental mutant.

Positive selection drugs were applied 72 h post-transfection, and media and drugs were refreshed every day for the first 7 days. The final concentrations of drugs were 2.5 μg/ml for BSD (Sigma), 2.5 nM for WR99210 (Sigma), and 1.5 μM for DSM1 (Phillips et al., 2008; Ganesan et al., 2011), respectively.

### Analysis of Transfected Parasites

Two weeks after electroporation, blood films were made from transfected cultures every 2–3 days. All the mutants were first verified by PCR analysis and DNA sequencing. For the verification, the infected red blood cells (iRBCs) were collected and lysed on ice with 0.15% saponin in phosphate-buffered saline (PBS). Genomic DNAs were extracted with TIANamp genomic DNA kit (DP304, Tiangen, China) according to the directions. Primers used for verifications are listed in Supplementary Table 1. PCR products were sequenced after being recovered from agarose gel and purified using TIANgel Midi Purification Kit (DP209, Tiangen, China).

### Western Blotting

Mutant parasites were harvested and lysed by 0.15% saponin in PBS on ice for 10–15 min. Released parasite cells were washed with PBS and centrifuged at 12,000 *g* for 5 min at 4°C several times until the supernatant was clear. Then the precipitated cells were resuspended in 1 × PBS and mixed with SDS-PAGE loading buffer. The mixture was boiled for 5 min and centrifuged at 12,000 *g* for 5 min. Proteins in the collected supernatant were separated on denatured polyacrylamide gels and transferred to NC membranes. The membranes were blocked with 5% skim milk in PBS for 2 h and incubated overnight at 4°C with diluted primary antibodies (mouse anti-Flag, 1:1000, sigma; mouse anti-HA, 1:1000, sigma; mouse anti-Ty1, 1:1000, sigma). The secondary antibody was horseradish peroxidase (HRP)–conjugated goat anti-mouse antibody (Abcam) at a dilution of 1:5000. Detection of target proteins was implemented with ECL (GE Healthcare) and results were recorded with a BG-gdsAUTO 710MINI imaging system (Baygene).

### Immunofluorescence Assay

Parasite cells were prepared from infected RBCs by 0.15% saponin treatment at 37°C for 5 min and fixed with fresh 4% paraformaldehyde in PBS on ice for 20 min. After three washes with PBS, parasites were deposited on microscope slides to air dry and incubated with 1% bovine serum albumin (BSA) in PBS for 1 h at room temperature. Then parasites were incubated with primary antibodies (mouse anti-Ty1, Sigma; rabbit anti-HA, Abcam) and diluted in 1% BSA-PBS for 1 h at room temperature. After three washes with PBS, parasites were co-incubated with the secondary Alexa 488 goat anti-mouse antibody (Thermo Fisher Scientific) and Alexa 568 goat anti-rabbit antibody (Thermo Fisher Scientific) at room temperature for 1 h and washed three times again with PBS. Finally, parasites were sealed with a mounting medium containing DAPI. Images were captured using a Nikon A1R confocal microscope.

### 0–3 h Ring-Stage Survival Assay

A total of 0–3 h ring-stage survival assay (RSA^0–3 h^) assay was performed as previously described (Witkowski et al., 2013). Parasites were synchronized several times to acquire the accurate 0–3 h post-invasion rings. The accurately synchronized ring-stage parasites were exposed to 700 nM dihydroartemisinin (DHA) or 0.1% dimethyl sulfoxide (DMSO, the solvent) for 6 h, washed with incomplete medium to remove the drug, and cultured for another 66 h. Blood films were made to assess the survival rates of the strains microscopically.

### PAM Analysis

We scanned the 3D7 reference genome for PAM-associated target sites. Unique genomic regions flanking PAMs (20 bp upstream NGG and 20 bp downstream TTTV) were regarded as possible target sites.

## Results

### Cas9 Expression Cassette Was Successfully Integrated Into the *P. falciparum* Genome

**FIGURE 2:**
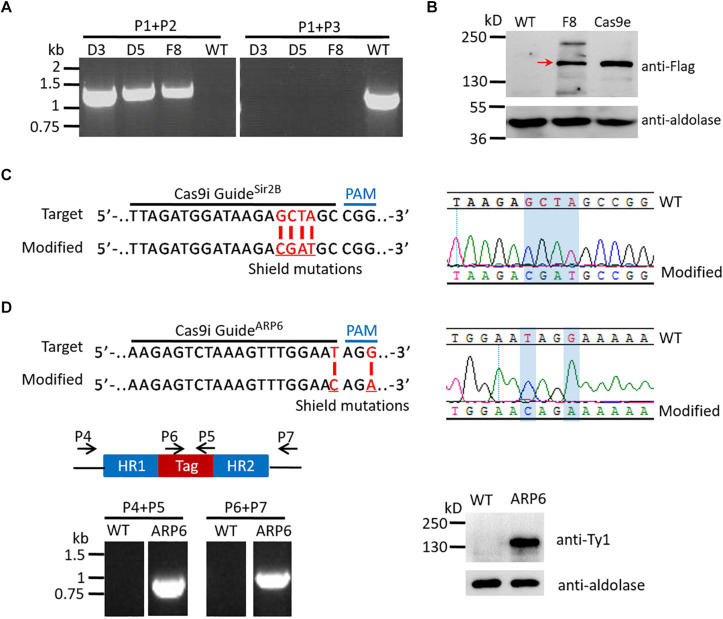
Examination of the parental Cas9i line and the mutants of single gene editing with it. **(A)** Analysis of Cas9i clone D3, D5, F8, and WT by PCR with primers P1–P3 indicated in Figure 1B. The templates were genomic DNAs of the corresponding strains. PCR analysis confirms the recombination locus is present in Cas9i clones (primers P1/P2, 1300 bp) and absent in WT, and the wild-type endogenous locus is absent in Cas9i clones and present in WT (primers P1/P3, 1390 bp). **(B)** Analysis of Cas9 protein in Cas9e and Cas9i by western blotting. We used the mouse anti-flag antibody to check the Cas9 protein and mouse anti-aldolase antibody to check the reference aldolase. The result shows the expression of the ∼156 kDa Cas9 protein in Cas9e and Cas9i (indicated by the red arrow). Cas9e, the mutant generated previously with Cas9e system; F8, the parental Cas9i clone F8. **(C)** Left: Target sequence for generation of *Sir2B-mut^*Cas9i*^* highlighting the 20-nt guide sequence and the PAM (top). Modified locus shows the shield mutations (red) (bottom). Right: DNA sequencing analysis of *Sir2B-mut^*Cas9i*^*. Desired shield mutations are highlighted. **(D)** Top left: Target sequence for generation of *ARP6-HA-Ty1^*Cas9i*^* highlighting the 20-nt guide sequence and the PAM (top). Modified locus shows the shield mutations (red) (bottom). Top right: DNA sequencing analysis of *ARP6-HA-Ty1^*Cas9i*^*. Desired shield mutations are highlighted. Bottom left: Diagram illustrating the primers used for PCR verification of *ARP6-HA-Ty1^*Cas9i*^* and WT (top), and the result of PCR analysis (bottom). PCR analysis confirms the presence and the absence of HA-Ty1 tag in *ARP6-HA-Ty1^*Cas9i*^* and WT respectively (primers P4/P5, 903 bp; primers P6/P7, 1076 bp). Bottom left: Western blotting of *ARP6-HA-Ty1^*Cas9i*^* and WT. We used the mouse anti-Ty1 antibody to check the ∼122 kDa ARP6-HA-Ty1 protein and the result shows the expression of ARP6-HA-Ty1. WT, wild-type 3D7.

The parental Cas9i line, in which the Cas9 expression cassette was integrated into genomic locus by a single-crossover recombination event (Collins et al., 2013), was generated to express stable Cas9 endonuclease, so that the *pUF1-Cas9* plasmid is no longer needed for electroporation when using the CRISPR/Cas9 system (Figure 1B). The *p230p* gene locus (PF3D7_0208900), which was reported to be a suitable site for integration without compromising parasite development during the blood-stage (Hughes and Waters, 2017; Knuepfer et al., 2017), was selected to generate the *pUF1-DHODH-Cas9i* plasmid for recombination. *pUF1-DHODH-Cas9i* was transfected into the *P. falciparum* 3D7 strain and selected with DSM1. Drug-resistant parasites, most of which were episomal ones, could be checked on Giemsa-stained blood films about 20 days after electroporation. And then the mutants proceeded to the DSM1 off/on cycles (3 weeks off and 2 weeks on) (Figure 1C) for stable expression screening. The integrative parasites were verified by PCR (using primer sets P1/P2 and P1/P3, as shown in Figure 1B) at the end of every cycle. The desired parasites appeared abundantly after two cycles. Limiting dilution was then done to obtain pure integrative monoclones. Eventually, three integrative Cas9i clones (D3, D5, and F8) were obtained and confirmed by PCR (Figure 2A). The ∼156 kDa Cas9 protein was expressed successfully and stably in Clone F8, which was detected by western blotting, with wild-type 3D7 and a transgenic mutant of Cas9e system as the negative and positive control, respectively (Figure 2B).

### Mutation or Tagging for a Single Gene Were Achieved With the Cas9i System

To test the gene editing efficiency of the Cas9i system, Sir2B (PF3D7_1451400), a transcriptional regulatory protein, and ARP6 (PF3D7_0719300), an actin-related protein, were selected to be edited in the parental Cas9i strain. In this study, all target sites in donor DNAs were mutated by several synonymous mutations as shield mutations, preventing repeated cleavage on modified loci by the endonuclease. For Sir2B, only synonymous shield mutations were introduced to minimize the corresponding pL6cs construct and to maximize the success rate of genome editing (Figure 2C). Besides the shield mutations, an HA-Ty1 tag was added to the C-terminal of ARP6. Two transgenic strains, *Sir2B-mut^*Cas*9*i*^* and *ARP6-HA-Ty1^*Cas*9*i*^*, were obtained. DNA sequencing indicated the successful introduction of shield mutations into the genomic loci of Sir2B (Figure 2C) and ARP6 (Figure 2D). PCR verified the correct insertion of the HA-Ty1 tag into ARP6 (using primers P4/P5 and P6/P7, Figure 2D). The ∼122 kDa ARP6-HA-Ty1 protein was correctly expressed, confirmed by western blotting (Figure 2D).

### Genome Editing Efficiency Was Significantly Improved With the Cas9i System

To confirm that the Cas9i system is more time-saving, four genes, Sir2B (PF3D7_1451400), ARP4 (PF3D7_1422800), ARP6 (PF3D7_0719300), and MRT4 (PF3D7_0602100) (Yin et al., 2020; see Table 1), were selected at random for editing by either Cas9i or Cas9e systems with the same pL6 constructs. Both approaches of site-mutation and tagging of target genes were examined by the two systems, since these are routine gene editing strategies for functional investigation of a given gene. Two tags with different sequence lengths were tested here: HA-Ty1 and Ty1-Ribo. The time intervals from transfection to the first appearance of resistant parasites for the transgenic strains edited by the Cas9e system were significantly higher than that edited by Cas9i system. For instance, the time interval of Sir2B strain was 29 days by the Cas9e system and 17 days by the Cas9i system. Overall, the maximum and average time for the resistant parasite to be detected were 29 days and ∼24.5 days in the Cas9e system, respectively, while in the Cas9i system, those were only 18 days and ∼16.5 days [*P* = 0.016 (unpaired two-tailed Student’s *t*-test)] (Table 1). This result confirms the significantly higher editing efficacy of the Cas9i system.

**TABLE 1:** Time for the first detection of transgenic parasites.

**Gene name**	**Gene ID**	**Gene edition**	**Construct**	**Tag length (bp)**	**Days**	
					**Cas9e**	**Cas9i**
Sir2B	PF3D7_1451400	Mutation	Shield mutations	–	29	17
ARP4	PF3D7_1422800	Knockin	ARP4-Ty1-Ribo	390	28	15
ARP6	PF3D7_0719300	Knockin	ARP6-HA-Ty1	219	21	16
MTR4	PF3D7_0602100	Knockin	MTR4-Ty1-Ribo	390	20	18
Average days*					24.5	16.5

*P = 0.016 (unpaired two-tailed Student’s *t*-test). Every gene was edited by two independent repeated transfections, and the average days of the two transfections are shown in the table.

### Dual Genome Editing Was Accessible in the Cas9i System

**FIGURE 3:**
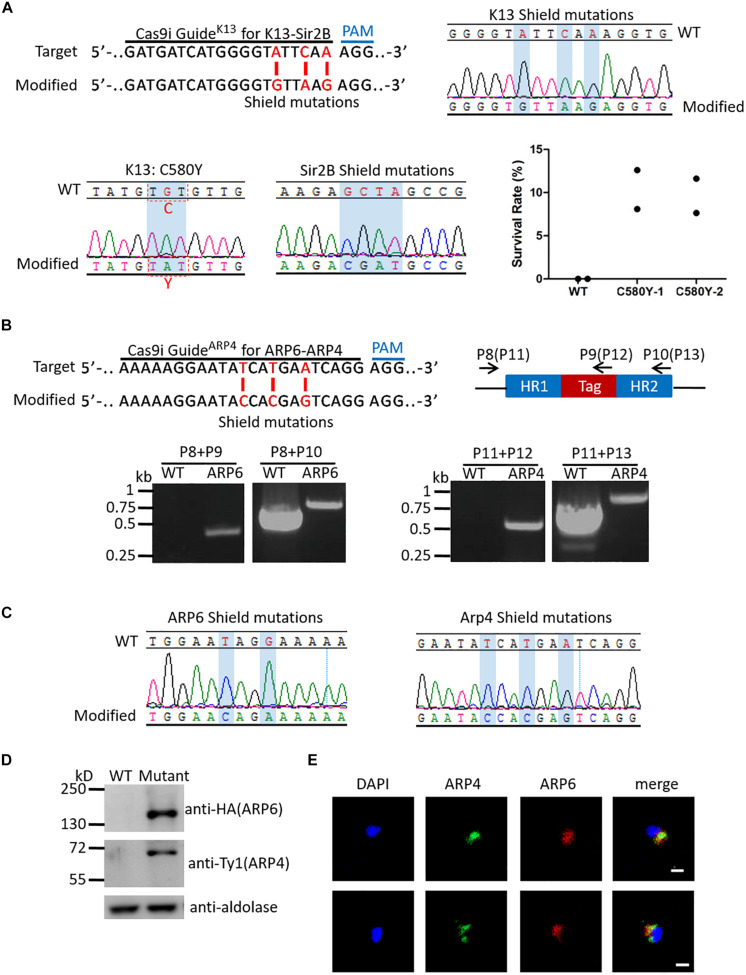
Examination of multiplex genome editing. **(A)** Top left: K13 target sequence for generation of *K13::Sir2B-mut^*Cas9i*^* highlighting the 20-nt guide sequence and the PAM (top). Modified locus shows the shield mutations (red) (bottom). Top right: DNA sequencing analysis of K13 shield mutations in *K13::Sir2B-mut^*Cas9i*^*. Desired shield mutations are highlighted. Bottom left: DNA sequencing analysis of K13_C580Y mutation in *K13::Sir2B-mut^*Cas9i*^*. C580Y mutation is highlighted. Bottom middle: DNA sequencing analysis of Sir2B shield mutations in *K13::Sir2B-mut^*Cas9i*^*. Desired shield mutations are highlighted. The 20-bp target and shield mutations for Sir2B are the same as those used in single gene editing. Bottom right: Survival rates of RSA^0– 3 h^ for *K13::Sir2B-mut^*Cas9i*^* clones and WT. Survival rates of mutant clones (mean 10.36, 9.62%) are significantly higher than WT (none), confirming the correlation between K13_C580Y mutation and artemisinin resistance. *n* = 2. WT, wild-type 3D7. **(B)** Top left: ARP4 target sequence for generation of *ARP6-HA::ARP4-Ty1^*Cas9i*^* highlighting the 20-nt guide sequence and the PAM (top). Modified locus shows the shield mutations (red) (bottom). The 20-bp target and shield mutations for APP6 are the same as those used in single gene editing. Top right: Diagram illustrating the primers used for PCR verification of *ARP6-HA::ARP4-Ty1^*Cas9i*^*. Primers for ARP6 (primers P8, P9, and P10) and ARP4 (primers P11, P12, and P13; in brackets) are indicated respectively. Bottom: PCR analysis of ARP6 (left) and ARP4 (right) in *ARP6-HA::ARP4-Ty1^*Cas9i*^* and WT. The result confirms the presence of HA tag in ARP6 (primers P8/P9, 446 bp in the mutant; primers P8/P10, 740 bp in the WT and 800 bp in the mutant) and Ty1 tag in ARP4 (primers P11/P12, 502 bp in the mutant; primers P11/P13, 738 bp in the WT and 810 bp in the mutant). **(C)** DNA sequencing analysis of shield mutations in *ARP6-HA::ARP4-Ty1^*Cas9i*^*. Desired shield mutations of ARP6 (left) and ARP4 (right) are highlighted. **(D)** Western blotting of *ARP6-HA::ARP4-Ty1^*Cas9i*^* and WT. We used the mouse anti-HA antibody to check the ∼116 kDa ARP6-HA protein and mouse anti-Ty1 antibody to check the ∼60 kDa ARP4-Ty1 protein. The result shows the expression of ARP6-HA and Arp4-Ty1 proteins. Mutant, *ARP6-HA::ARP4-Ty1^*Cas9i*^*. **(E)** IFA analysis of *ARP6-HA::ARP4-Ty1^*Cas9i*^*. Nuclei were stained with Hoechst 33342 (blue fluorescence). The green fluorescence represents ARP4-Ty1 and the red fluorescence represents ARP6-HA. IFA reveals the co-localization of ARP4 and ARP6 in the nucleus. Bar = 5 μm.

Mutations of K13 (Kelch propeller protein Kelch 13, PF3D7_1343700) was reported to be the marker of resistance to artemisinin and its derivatives in *P. falciparum* (Ariey et al., 2014). Among them, K13_C580Y is the most typical mutation (Straimer et al., 2015). The K13_C580Y mutation (with shield mutations) and the Sir2B shield mutations were introduced into the Cas9i system by plasmid *pL6cs-K13-Sir2B*. The desired transgenic strain *K13::Sir2B-mut^*Cas*9*i*^* was obtained in about 20 days. To identify *K13::Sir2B-mut^*Cas*9*i*^*, clones were identified by limiting dilution, and DNA sequencing indicated the correct introduction of K13_C580Y mutation and shield mutations (Figure 3A). To assess the level of artemisinin resistance, RSA^0–3 h^ was performed. The result showed that C580Y-mutated clones have a survival rate of ∼10% on average, while nearly no parasites survived in wild-type 3D7 (Figure 3A), which was consistent with previous reports (Ghorbal et al., 2014).

ARP4 (PF3D7_1422800), like ARP6, is another member of actin-related proteins (Liu et al., 2020). The homology proteins of ARP4 and ARP6 in yeast are both components of the same chromatin remodeling complex (Yoshida et al., 2010). To explore if these two proteins have similar functions in *P. falciparum*, an HA tag and a Ty1 tag were added to the C-terminal of ARP6 and ARP4, respectively, and were introduced into the Cas9i system by plasmid *pL6cs-ARP6-ARP4*. These failed in the Cas9e system. The desired strain *ARP6-HA::ARP4-Ty1^*Cas*9*i*^* was verified by PCR (primer sets P8/P9 and P8/P10 for ARP6, primer sets P11/P12 and P11/P13 for ARP4, Figure 3B) and the result revealed that the tags were inserted correctly. Subsequent DNA sequencing showed the successful introduction of shield mutations (Figure 3C). Next, western blotting confirmed the successful expressions of the ∼116 kDa ARP6-HA protein and the ∼60 kDa ARP4-Ty1 protein (Figure 3D). Accordingly, an immunofluorescence assay (IFA) was performed to check the co-localization of ARP6 and ARP4 in parasite cells. The result showed the two proteins co-localized in the nucleus (Figure 3E), which indicated that ARP4 and ARP6 in *P. falciparum* have similar functions to those in yeast.

### TTTV PAM of AsCpf1 Is More Competitive for Gene Editing in *P. falciparum*

**FIGURE 4:**
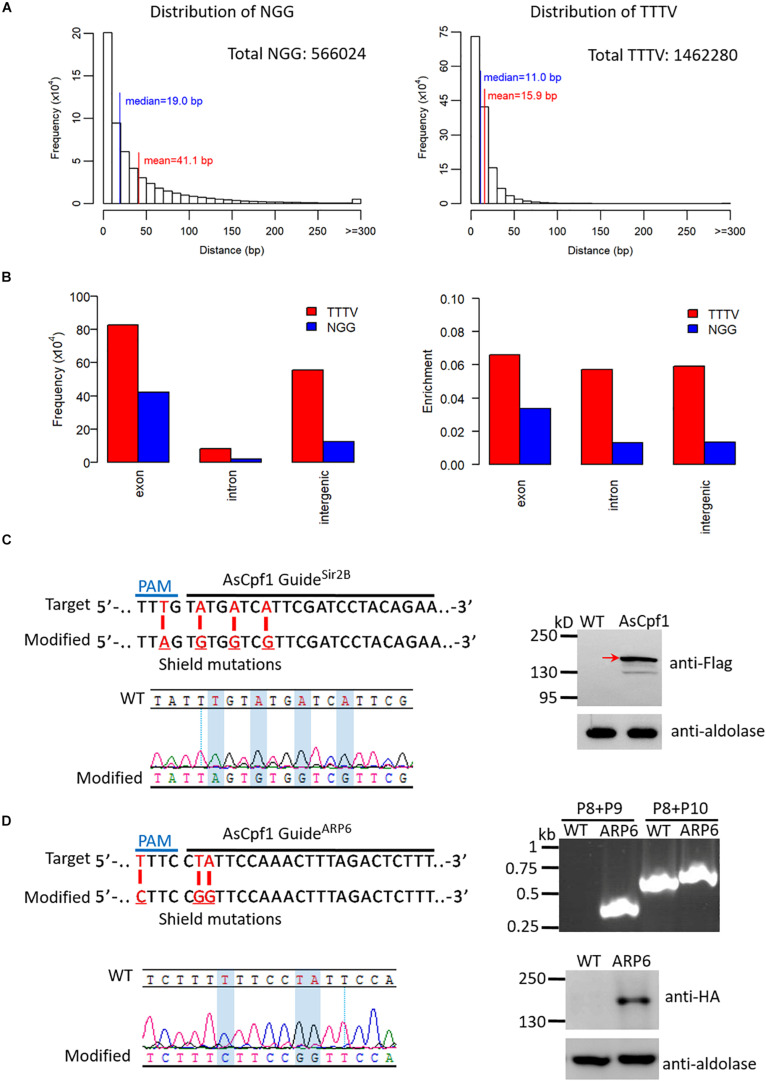
Adaptation of AsCpf1 in *P. falciparum*. **(A)** Histogram of distances between adjacent NGGs (left) and TTTVs (right) with unique 20-bp target sites in the 3D7 genome. The horizontal axis represents the distance between two adjacent NGGs or TTTVs, and the vertical axis represents the frequency of NGG or TTTV at this distance throughout the genome. The median and mean distances, as well as the total number of possible target sites for NGG and TTTV, are shown. **(B)** Distribution analysis of NGG and TTTV in distinct regions of the 3D7 genome. The frequency (left) and enrichment (right) of NGG and TTTV in exon, intron, and intergenic regions are shown. The level of enrichment is shown as the ratio of the frequency to the size of the region. **(C)** Top left: Target sequence for generation of (*Sir2B-mut^*AsCpf1*^* highlighting the 23-nt guide sequence and the PAM (top). Modified locus shows the shield mutations (red) (bottom). Bottom left: DNA sequencing analysis of *Sir2B-mut^*AsCpf1*^*. Desired shield mutations are highlighted. Right: Detection of AsCpf1 protein in *Sir2B-mut^*AsCpf1*^* by western blotting. We used a mouse anti-flag antibody to check the ∼150 kDa AsCpf1 protein and the result shows its expression (red arrow). **(D)** Top left: Target sequence for generation of *ARP6-HA^*AsCpf1*^* highlighting the 23-nt guide sequence and the PAM (top). Modified locus shows the shield mutations (red) (bottom). Top right: PCR analysis of *ARP6-HA^*AsCpf1*^* and WT. Primers are indicated in Figure 3B (primers P8, P9, and P10). The result confirms the presence of HA tag in ARP6 (primers P8/P9, 446 bp in mutant; primers P8/P10, 740 bp in WT, and 800 bp in the mutant). Bottom left: DNA sequencing analysis of *ARP6-HA^*AsCpf1*^*. Desired shield mutations are highlighted. Bottom right: Western blotting of *ARP6-HA^*AsCpf1*^* and WT. We used a mouse anti-HA antibody to check the ∼116 kDa ARP6-HA protein and the result shows its expression.)

AsCpf1, a Cpf1 ortholog confirmed to be active in mammalian cells, is guided by a single crRNA bearing a 19-nt DR and a 23-nt target sequence, and it employs a TTTV PAM (V can be A, G, or C) (Supplementary Figure 1). Considering the AT-rich genome of malaria parasites, the distributions of NGG PAMs for SpCas9 and TTTV PAMs for AsCpf1 throughout the *P. falciparum* 3D7 reference genome were analyzed. For NGG PAM, there were 566,024 possible target sites with an average distance of ∼41 bp. While for TTTV PAM, there were 1,462,280 possible target sites with an average distance of ∼16 bp (Figure 4A). This indicates that there are more editable genomic sites for AsCpf1 on the AT-rich genome. Based on the analysis of the genomic distribution of these PAM-associated target sites, there are more candidate target sites for TTTV PAM than for NGG PAM in exon, intron, and intergenic regions (Figure 4B). In the exon region specifically, both the frequency and the abundance of TTTV are almost twice that of NGG, which demonstrates that AsCpf1 can recognize more cleavage sites and could be more competitive for genome editing in *P. falciparum* than SpCas9.

### Genome Editing With AsCpf1 in *P. falciparum*

Similar to the Cas9i system, AsCpf1 was tested by mutating Sir2B or tagging ARP6, and the transgenic mutants *Sir2B-mut^*AsCpf*1^* and *ARP6-HA^*AsCpf*1^* were generated, respectively. Sir2B with shield mutations was confirmed to be edited correctly by sequencing (Figure 4C). In addition, the western blotting analysis revealed the successful expression of the ∼150 kDa AsCpf1 protein in *Sir2B-mut^*AsCpf*1^* (Figure 4C). For ARP6, the HA tag fused to the C-terminal of ARP6 was verified by PCR, and shield mutations were confirmed correctly by sequencing (Figure 4D). The ∼116 kDa ARP6-HA protein was confirmed to be expressed correctly by western blotting (Figure 4D). Thus, two types of gene editions by mutating or tagging with AsCpf1 in *P. falciparum* have been successfully realized. This provides an alternative gene editing tool for those target genes without suitable PAMs for the usage of current Cas9 system.

## Discussion

Due to the special parasitic status, the study of gene function based on gene modification in *Plasmodium* parasites has long been restricted by the extremely rare and inefficient genome editing technologies. Most traditional genome editing methods are based on the single- or double-crossover of homologous arms taking advantage of homologous recombination, which may require several time-consuming drug off/on cycles. Moreover, recombination occurs at a random site of the homologous arms, which may result in invalid editing. So more efficient and specific genome editing tools, such as ZFN and TALEN (Boch et al., 2009; Moscou and Bogdanove, 2009), which are adapted well in other organisms, are expected to be useful in malaria parasites. ZFN has been successfully adapted to *P. falciparum* parasites (Straimer et al., 2012; McNamara et al., 2013), but is still restricted by the limited target sites and high cost, while nearly no studies have reported the successful application of TALEN in *P. falciparum* up to now.

The CRISPR/Cas system has made genome editing simpler and more accurate in various organisms, including in malaria parasites. In this study, we developed an integrating strategy and generated a Cas9i system to facilitate the utilization of Cas9 in *P. falciparum* parasites. Qian et al. (2018) reported a method to generate a similar Cas9-knock-in mutant in rodent malaria parasite *Plasmodium yoelii*, which is inaccessible in *P. falciparum* because of the excessively large constructs. Thus, in this study, we generated the Cas9i line through traditional single-crossover recombination and drug off/on cycles. The Cas9i system remarkably increases the success rate of editing and shortens the time for selection of transgenic parasites, and it can greatly improve the efficiency of genome editing by multiplexed genome editing when the interaction of distinct genes is to be studied. Therefore, the Cas9i system can significantly aid in the study of immune evasion and host-parasite interaction, such as the regulation mechanism of var expression and drug resistance, which probably involve the interaction of two or more proteins.

We have also attempted to edit three genes simultaneously, but only the first target was modified (data not shown), perhaps owing to the too-long sgRNA array. In addition, besides the 3D7 strain, this integrating strategy can be applied in many other strains if necessary. And a more time-saving method, termed selection-linked integration (SLI), which was introduced by Birnbaum et al. (2017) for quick selection of genomic integration, can be used instead of the time-consuming drug off/on cycles. With this SLI method, the integrating strategy can be conveniently adapted to many other engineered CRISPR endonucleases. For example, Zetsche et al. (2015b) developed a split-Cas9 system, in which Cas9 is split into two fragments and fused to FK506 binding protein 12 (FKBP) and FKBP rapamycin binding (FRB) to take advantage of the FKBP-rapamycin-FRB ternary complex, achieving inducible genome editing and transcription modulation in human cells. Furthermore, Komor et al. (2016) fused a cytidine deaminase enzyme to catalytically dead Cas9 (dCas9) to mediate the direct conversion of cytosine (C) to thymine (T) without inducing double-stranded DNA breaks in mammalian cells, and subsequently, this strategy was adapted to Cpf1 (Li et al., 2018). Because of the complicated constructs, it would be inevitably inefficient to utilize these derived tools in malaria parasites with an episome-based strategy, in which case the integrating strategy provides a better choice.

Due to the AT-rich genome of *P. falciparum*, the utilization of Cas9 is restricted by the G-rich PAM NGG, therefore we adapted the CRISPR/Cpf1 system employing the T-rich PAM TTTV and generated two genome-edited transgenic mutants with this system. As far as we know, this demonstration of genome editing with Cpf1 is the first in malaria parasites. Because of the lack of enzymes for non-homologous end joining (NHEJ), the double-strand breaks (DSBs) of DNA are repaired mainly by homologous recombination in malaria parasites. Therefore, few parasites with off-target DNA cleavage by Cas9 would survive, which contributes to the lack of evidence suggesting off-target activity of Cas9. In contrast, besides from the dominance of T-rich PAM sites, Cpf1 cleaves double-strand DNA target in a staggered way (Supplementary Figure 1), and the resulted staggered ends can be repaired by parasites through microhomology-mediated end joining (MMEJ) without introducing indels (Xu et al., 2019). Thus, more parasites with off-target DNA cleavage would survive owing to the staggered cut, which will make Cpf1 more efficient in malaria parasites. In addition, AsCpf1 employs a much shorter 19-nt DR and possesses the ability to process its own crRNA, providing more convenience for multiplexed genome editing with crRNA array, although we failed in multiplexed genome editing with AsCpf1, perhaps owing to the low efficiency of the episome-based strategy. In that case, the integrating strategy would further facilitate the application of Cpf1 in *Plasmodium* parasites.

## Conclusion

In summary, we have developed an integrating strategy for CRISPR/Cas9 and generated a parental Cas9i line in *P. falciparum*. With this line, we have obtained a variety of single gene-edited transgenic strains and two transgenic strains with double genes edited simultaneously, demonstrating how this Cas9i system has the advantages of being less time-consuming and having a higher success rate. Additionally, we adapted the CRISPR/Cpf1 system in *P. falciparum* for the first time, which could be a potential alternative to Cas9 for genome editing in malaria parasites. We hope these optimizations of the CRISPR/Cas system for genome editing can greatly aid in the study of gene function and molecular mechanism in *P. falciparum* and contribute to the elimination of malaria.

## Data Availability Statement

The original contributions presented in the study are included in the article/Supplementary Material, further inquiries can be directed to the corresponding author/s.

## Author Contributions

QZ and LS conceived and designed the experiments. YZ, FW, and CW generated transgenic parasite lines and related analysis. XZ and CJ performed the informatics analysis. QZ, LS, and YZ wrote the manuscript with contribution from FD. All authors read and approved the final manuscript.

## Conflict of Interest

The authors declare that the research was conducted in the absence of any commercial or financial relationships that could be construed as a potential conflict of interest.

## Supplementary Material

Supplementary Figure 1 Diagram illustrating target DNA substrate recognized by SpCas9 sgRNA or AsCpf1 crRNA. The cleavage sites on both strands are indicated (blue triangles).
