# Supplementary figures and images for: Optimization of CRISPR/Cas System for Improving Genome Editing Efficiency in Plasmodium falciparum
Source: Front Microbiol. 2021 Jan 8;11:625862. doi: 10.3389/fmicb.2020.625862 (PMC7819880; doi:10.3389/fmicb.2020.625862)

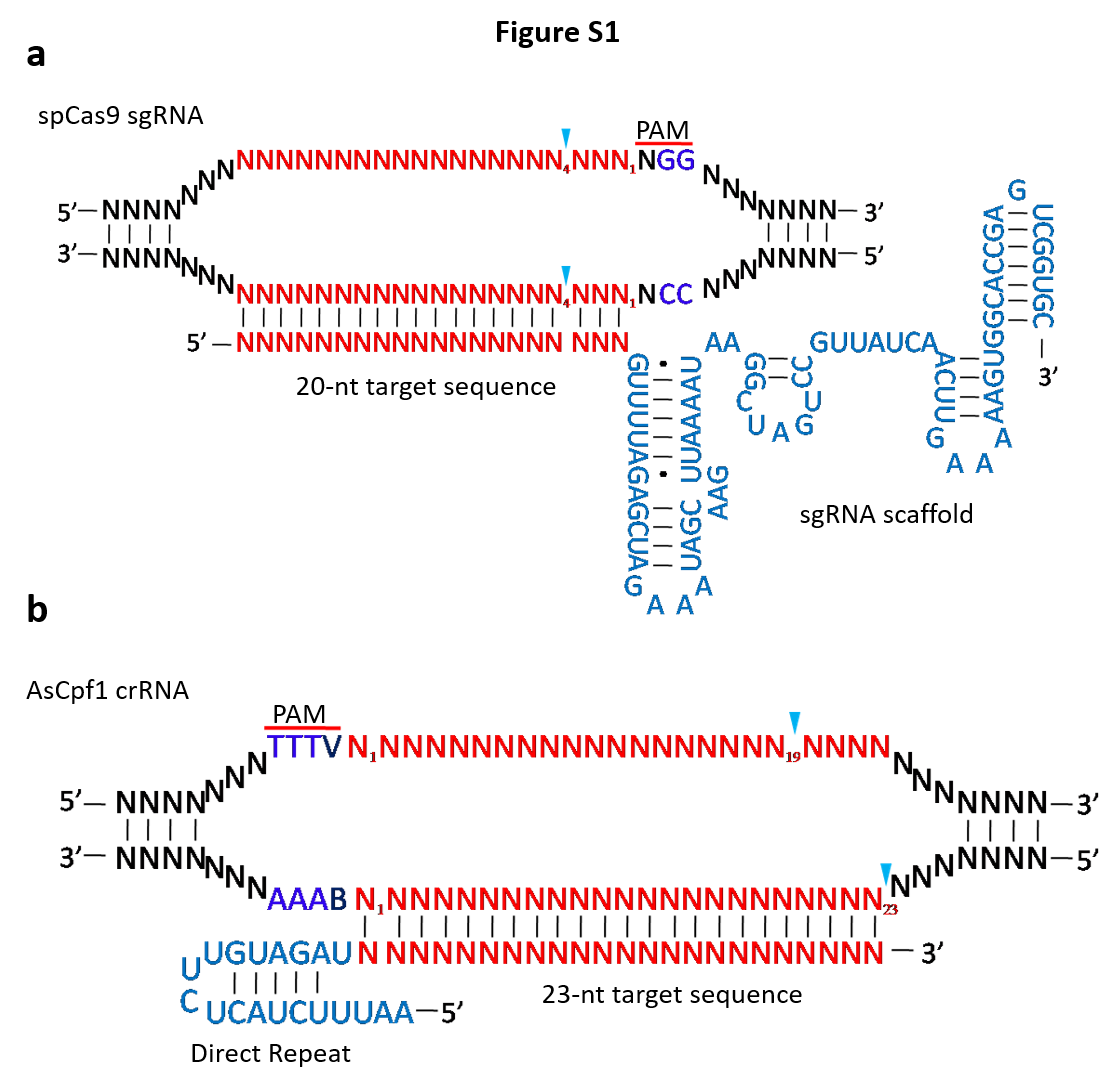

Supplement: Supplementary Figure 1 — Diagram illustrating target DNA substrate recognized by SpCas9 sgRNA or AsCpf1 crRNA. The cleavage sites on both strands are indicated (blue triangles). [file Image_1.TIF]
